# Military Personals

**Published:** 1918-02

**Authors:** 


### MILITARY PERSONALS

---

To Army Medical School, Washington, D. C., Major Frederick N. Jerauld, Niagara Falls.

To Boston, Mass., Lieut. Walter J. Smith, Waterville.

To Camp Cody, Deming, N. M., Lieut. Leon H. Prior.

To Camp Devens, Ayer, Mass., Lieut. Albert Bowen, Rochester.

To Camp Greene, Charlotte, N. C., Major W. Brewer, Geneva.

To Camp Meade, Anapolis Junction, Md., Lieut. Lee A. Hadley, Syracuse.

To Camp Wheeler, Macon, Ga., Lieut. Maxwell K. Willoughby, Auburn.

To Fort Riley, Kan., Capt. Robert King, Buffalo.

To New York City, Lieuts. Ira W. Livermore, Gowanda; Chas. Jennings, Elmira; Michael Schultz, Astoria.

To Philadelphia, Pa., Capt. Harry B. Huver, Williamsville; Lieut. Alexander L. Smith, Rochester.

To Camp Custer, Battle Creek, Mich., Capt. Lynn S. Beals, Buffalo.

To Camp Upton, L. I., Capt. Max Einhorn, New York City.

To Fort Ogelthorpe, Ga., Capt. William B. Reid, Rome; Lieuts. John H. Watson, Joseph L. Wozniak, Buffalo; Frederick B. Bond, Prattsburg.

To Fort Sill, Okla., Lieut. Edwin S. Ingersoll, Rochester.

Lt. Benj. J. Slater of Rochester, to Army Med. School, Washington, from Camp Lee.

Lt. Edward F. Meister of Buffalo, to Aviation Section at Buffalo, to examine recruits.

Capt. Chas. E. Long of Buffalo, to Camp Hancock, Augusta, Ga., motor mechanic regiments, from Camp Sheridan, Ala.

Lt. Edmund B. Spaeth of Elmira, to Base Hospital, Camp Meade, Md.

Lt. A. C. Smith of Elmira, to Ft. Oglethorpe, Ga., for instruction.

Lt. Francis J. Haley of Buffalo, to Ft. Porter with Hospital Unit F.

Capt. C. K. Haskell of Rochester, to N. Y. Post-Graduate Medical School for instruction.

Capt. Roland O. Meisenbach of Buffalo to the Base Hospital, Camp Lee, Petersburg, Va.

Lt. Raymond J. DeVine, Syracuse, to Ft. Foster, N. Y., for duty.

Major Edward A. Southall of Buffalo to Ft. Oglethorpe, for instruction.

Lt. Geo. E. Stevenson of Himrods to Ft. Slocum, N. Y., for duty, from Ft. Ethan Allen.

Lt. Geo. B. Ubel of Salamanca, to Washington University, St. Louis, for instruction in Urology, from Camp Jackson.

Dr. Cyril G. Hutchinson of the British and Canadian Recruiting Mission has been transferred from duty at Buffalo to Rochester. He is succeeded by Capt. R. F. Kee, R. A. M. C., who has seen active service on the western front and at Gallipoli.

Capt. Harry B. Huver of Williamsville has been transferred from the Evans Dental Inst. at Philadelphia to the Base Hospital at Camp Jackson, Columbia, S. C.

Lt. A. H. Vogt of Buffalo to Camp McClellan, Anniston, Ala.

Lt. John C. Brady of Buffalo, to Ft. McPherson, Ga., Hospital Unit H.

Lt. Vernon L. Bishop of Livonia to Camp Lewis, Wash., as sanitary inspector.

Capt. S. A. Mahady of Utica to Camp Meade, Annapolis Junction, for duty in Base Hospital.

Capt. Raymond C. Conklin, Buffalo, to Base Hospital, Camp Taylor, Louisville, Ky.

Lt. Geo. B. Ubel of Salamanca, to Ft. Logan H. Roots, Ark., for temporary duty.

Lts. L. M. Belzer, Buffalo, Guy Granger of Sherman, to Ft. Oglethorpe, Ga., for instruction.

Lts. Peter J. Barone of Buffalo and Geo. K. Smith of Syracuse, to Hoboken, N. J.

Lt. Otto E. Utzinger of Ithaca, to University of Pa., for instruction.

Lts. Theron B. Bond of Cuba and J. A. Murphy of Buffalo, to Rochester, Minn., for instruction.

Lt. Ralph E. Robinson of Belmont, to Rockefeller Inst., for instruction.

Lt. Paul E. Betowski of Bath, to San Antonio, Tex.

